# Immune Dysfunction in Uremia—An Update

**DOI:** 10.3390/toxins4110962

**Published:** 2012-10-24

**Authors:** Gerald Cohen, Walter H. Hörl

**Affiliations:** Abteilung für Nephrologie und Dialyse, Univ.-Klinik für Innere Medizin III, Währinger Gürtel 18-20, Wien A-1090, Austria; Email: walter.hoerl@meduniwien.ac.at

**Keywords:** cardiovascular disease, oxidative stress, inflammation, infection, priming, apoptosis, uremic toxins, polymorphonuclear leukocytes, monocytes, antigen-presenting cells

## Abstract

Kidney dysfunction leads to disturbed renal metabolic activities and to impaired glomerular filtration, resulting in the retention of toxic solutes affecting all organs of the body. Cardiovascular disease (CVD) and infections are the main causes for the increased occurrence of morbidity and mortality among patients with chronic kidney disease (CKD). Both complications are directly or indirectly linked to a compromised immune defense. The specific coordinated roles of polymorphonuclear leukocytes (PMNLs), monocytes/macrophages, lymphocytes and antigen-presenting cells (APCs) in maintaining an efficient immune response are affected. Their normal response can be impaired, giving rise to infectious diseases or pre-activated/primed, leading to inflammation and consequently to CVD. Whereas the coordinated removal via apoptosis of activated immune cells is crucial for the resolution of inflammation, inappropriately high apoptotic rates lead to a diminished immune response. In uremia, the balance between pro- and anti-inflammatory and between pro- and anti-apoptotic factors is disturbed. This review summarizes the interrelated parameters interfering with the immune response in uremia, with a special focus on the non-specific immune response and the role of uremic toxins.

## 1. Magnitude of the Problem: Mortality in Uremia

**Figure 1:**
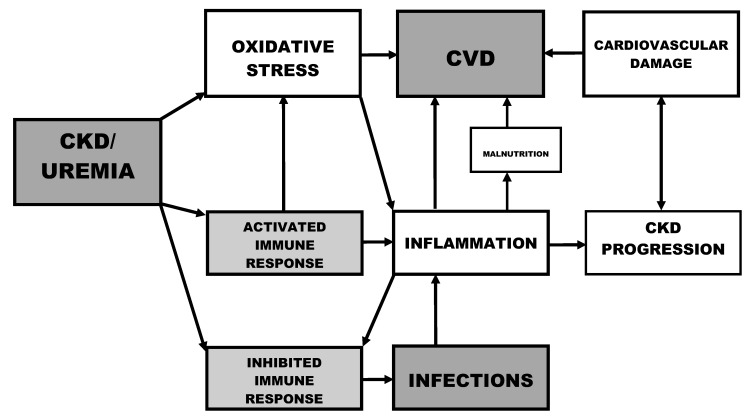
Immune dysfunction and risk factors in chronic kidney disease.

The development of CKD is associated with a significant increase in all-cause mortality [1]. As compared to the general population, a 2-fold incidence of mortality of CKD patients over 65 years and a 36-fold increase in mortality of CKD patients aged 16 to 49 years has been reported [2]. The main factors responsible for the increased risk of morbidity and mortality in patients with CKD are CVD and infections [3,4]. Both complications are linked to a disturbed immune response (Figure 1). In uremia, a diminished immune defense contributes to the high prevalence of infections, whereas pre-activation and priming of immune cells lead to inflammation and consequently to CVD. Nowadays, the term “uremia” describes the illness in renal failure, largely due to the retention of substances normally cleared by the kidneys [5]. 

Deranged functions of PMNLs contribute to the increased risk of bacterial infections and represent a main cause for the enhanced risk of morbidity and mortality among CKD patients [6]. PMNLs, cells of the first-line nonspecific immune defense, migrate to the site of infection along a chemotactic gradient; they ingest the invading microorganisms by phagocytosis and kill them with proteolytic enzymes and toxic oxygen radicals produced during the oxidative burst. Disturbances of any of those essential PMNL functions give rise to an increased risk for bacterial infections. The susceptibility of CKD patients to infections as a result of defective phagocytosis is caused by a variety of factors, including uremic toxins, iron overload, anemia of renal disease and dialyzer bioincompatibility [7].

Patients with CKD not on dialysis have an increased risk of bloodstream infection associated with an estimated glomerular filtration rate (GFR) less than 30 mL/min/1.73 m^2^ [8]. Furthermore, dialysis patients have higher annual mortality rates caused by sepsis compared with the general population, even after stratification for age, race and diabetes mellitus [9]. Another factor predisposing to infections is an inadequate response to vaccinations as a result of a deficient *T*-lymphocyte-dependent immune response [10]. 

CVD is the main cause of morbidity and mortality in patients with renal failure [11]. Cardiac failure is more common in CKD patients than in the general population [12] and is an independent predictor of death in CKD [13]. Cardiovascular risk factors and kidney function change concurrently, resulting in an increased risk of CVD as kidney function worsens [14]. This graded risk of cardiovascular mortality with decreasing GFR increases distinctly at an estimated GFR < 45 mL/min/1.73 m^2^ [15]. 

## 2. Oxidative Stress and Inflammation

Oxidative stress and inflammation are crucial for the defense against infections, but they initiate a number of deleterious effects if not properly regulated [16]. Oxidative stress increases in parallel with the progression of CKD and correlates with the level of renal function [17] (Figure 1). Furthermore, the antioxidant systems are severely impaired in CKD patients and worsen progressively with the degree of renal failure [18].

Biomarkers for oxidative stress, such as advanced oxidation protein products (AOPPs) and myeloperoxidase (MPO)-activity, and for inflammation, such as high sensitivity C reactive protein and interleukin (IL)-6, are interrelated in CKD [19]. The chronically activated immune system in uremia leads to a chronic low-grade inflammation, and consequently to atherosclerotic CVD [20]. Activated phagocytes represent a link between oxidative stress and inflammation [21] (Figure 1). Monocytes and PMNLs recognize pathogens via toll-like receptors (TLRs), inducing cellular activation and secretion of inflammatory cytokines. Monocyte TLR2 and TLR4, PMNL TLR4 expressions and TLR4 activity are elevated in hemodialysis patients, coupled with increased cytokine production in response to TLR4 activation with lipopolysaccharide [22]. Their scavenger receptor (CD36) processes oxidized lipoproteins and is a key modulator of proinflammatory and oxidative pathways [23]. Glycation and oxidation markers are simultaneously increased in uremia, e.g., in peritoneal patients where a correlation between markers of oxidative stress and advanced glycation end-products (AGE) concentrations has been reported [24]. The accumulation of protein damage products and their scavenger receptor-dependent recognition may represent a basic event in the establishment of a vicious and self-propelled “inflammatory loop” [25].

Persistent inflammation, *per se*, is a risk factor for the progression of CKD [26,27] (Figure 1) and may modulate the impact of other vascular and nutritional risk factors in the toxic uremic milieu [28]. Therefore, reducing inflammation may provide a novel means for treating kidney disease [29,30]. Chronic inflammation may cause malnutrition, itself an important risk factor for the development of CVD [31] (Figure 1). The tendency of CKD patients to develop CVD is accompanied by particular metabolic changes, e.g., in lipid profile and in homocysteine (Hcy) and C reactive protein serum levels [32]. The causes of inflammation are multifactorial, including patient-related factors, such as oxidative stress and infections, and hemodialysis (HD)-related factors such as biocompatibility and dialysate quality [33]. There is a correlation between the presence of bacterial DNA in dialysate and the increase in oxidative stress and serum levels of high sensitivity C reactive protein and IL-6 [34]. PMNLs of HD patients with a low level of pre-dialysis plasma bicarbonate concentration have a low intracellular pH level that may contribute to increased oxidative burst reactions [35]. 

## 3. Priming of Immune Cells

Priming of leukocytes is an important physiological mechanism controlling host defense responses, leading to a continuum of activation states [36]. Nevertheless, priming is an often overlooked and misinterpreted feature of immune cells. During priming, the functional response to a stimulus is amplified by previous exposure to a priming agent. PMNL activities can be primed by a transient rise in intracellular calcium concentrations [Ca^2+^]_i_ [37]. Priming of PMNLs influences their survival by attenuating constitutive apoptosis [38]. The oxidative burst of uremic PMNL can return to a non-primed state in the presence of normal plasma [39] and spontaneously fully “de-prime” after an initial challenge with platelet-activating factor [40], thus reversible priming leads to a so-far unrecognized flexibility in the modulation of PMNL function at sites of inflammation.

**Figure 2:**
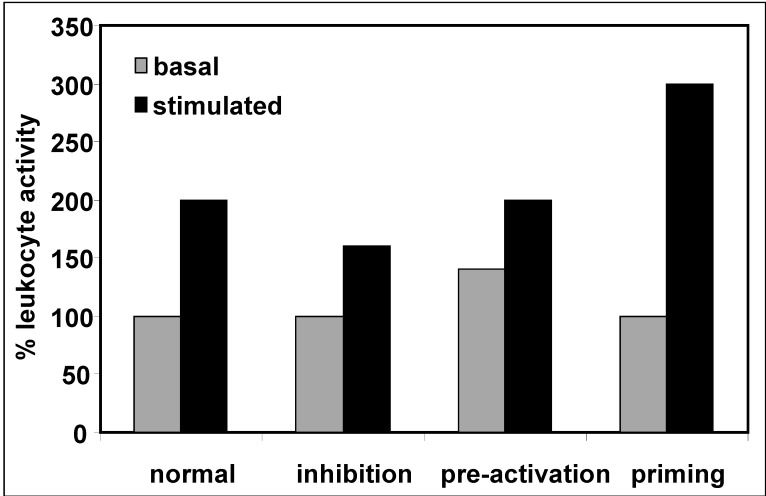
Different activation states and conditions influencing leukocyte function upon stimulation.

In diseases with a compromised immune defense such as uremia, the activation of immune cell functions by factors such as invading microorganisms can be inhibited, pre-activated at baseline or primed (Figure 2). An inhibited stimulation reflects a diminished immune response, and potentially leads to infections, whereas pre-activation where the basal activation state is elevated may cause inflammatory complications (Figure 1). 

Inappropriate PMNL priming is a key mediator of low-grade inflammation and oxidative stress in CKD patients observed before the onset of renal replacement therapies [41]. The side effects of dialysis treatment and the accumulating uremic toxins are main players leading to inadequate priming, and thereby to the induction of a vicious circle of oxidative stress and inflammation in CKD [26]. The incubation of normal PMNLs in plasma from patients with CKD or in peritoneal dialysis (PD) effluent primes and stimulates the production of reactive oxygen species, suggesting that priming involves a factor(s) retained in plasma as a result of renal failure. Hemodiafiltration (HDF) partially normalizes the PMNL oxidative burst, whereas HD does not [42], suggesting that HDF allows reduction of factors that may impair the oxidative burst.

## 4. Apoptosis

Since the immune deficiency in CKD coexists with the activation of immune cells, contributing to chronic inflammation [27,31], the coordinated removal of PMNLs is important for the resolution of inflammation. Enhanced apoptosis causes a diminished immune response, whereas delayed PMNL apoptosis or impaired clearance of apoptotic PMNLs by macrophages leads to an inflammatory state [43,44]. Therefore, the maintenance of a balance between anti-apoptotic and pro-apoptotic factors is essential [45]. The micro-environment and the local concentrations of PMNL modulating substances have to be considered. Extracellular acidosis inhibits PMNL apoptosis [46]. The intracellular acidification of PMNLs in HD patients with low plasma bicarbonate concentrations may contribute to delayed apoptosis [35].

We previously characterized free immunoglobulin light chains (IgLCs) as PMNL apoptosis inhibiting proteins [47]. Glucose-modified proteins are apoptosis-promoting factors [48], whereas phenylacetic acid [49] and *p*-hydroxy-hippuric acid [50], an erythrocyte plasma membrane Ca^2+^-ATPase inhibitor accumulating in uremic sera [51], attenuate PMNL apoptosis. The complement factor C5a also delays apoptosis of human PMNLs via phosphoinositide-3 kinase [52] and the ERK-signaling pathway [53]. Apoptosis of aging PMNLs depends on a superoxide release-dependent pathway, whereas tumor necrosis factor alpha (TNFα)-induced apoptosis seems to be unrelated to respiratory burst oxidase activity [54]. A temporary increase in [Ca^2+^]_i _acts as an important second messenger in PMNLs [55,56], leading to the modulation of apoptotic cell death [54,57,58].

Monocytes from dialysis patients exhibit characteristics of senescent cells, related to an increased susceptibility to apoptosis as demonstrated *in vitro* [59]. In HD patients—but not in continuous ambulatory peritoneal dialysis patients—there is an association between increased monocyte apoptosis and a decreased intracellular pool of thiols [60]. 

B lymphocytes of pre-dialysis CKD and HD patients have a higher rate of apoptosis than healthy controls. This increased susceptibility to apoptosis may contribute to B lymphopenia in CKD [61]. T cells from CKD patients have an aberrant state of early activation. Activated T cells may be driven to apoptosis, thereby contributing to T lymphopenia, progressive immunodeficiency and increased infection risk seen in these patients [62].

Dialysis normalizes the increased PMNL apoptosis rates observed in CKD patients [63], and lymphocyte apoptosis was greater in patients on low-flux than on high-flux membranes [64]. Both findings suggest the existence of dialyzable factors that modulate PMNL apoptosis. Monocyte apoptosis in uremia can be normalized by continuous blood purification methods such as PD, which may have advantages over intermittent therapies in removing uremic apoptotic molecules [65].

## 5. Metabolic Kidney Activities

**Figure 3:**
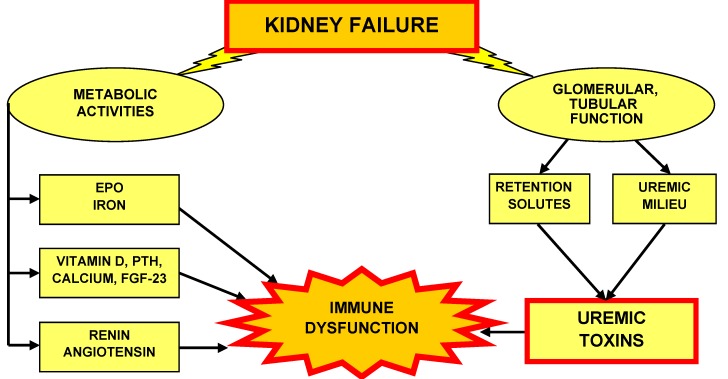
Kidney failure leads to disturbed renal metabolic activities and to impaired glomerular filtration and/or tubular secretion/reabsorption.

Some uremia related defects are reversed by transplantation, but not by dialysis treatment. This implies that besides impaired glomerular filtration, disturbed parenchymal metabolic activities of the kidney may be involved [66]. The hormone erythropoietin (EPO), the vitamin D receptor activator calcitriol (1,25(OH)_2_D_3_) and the enzyme renin are examples for renal produced substances affecting the immune system (Figure 3).

### 5.1. Erythropoietin and Iron

EPO is a hormone produced by the kidney and involved in the formation of red blood cells in the bone marrow. Patients with CKD have lower EPO serum levels than subjects with normal kidney function. Not all patients respond adequately to long-term treatment with recombinant human EPO. Resistance to recombinant human EPO can be caused by chronic inflammation, which can modify erythropoiesis via pro-inflammatory cytokines, such as IL-1, TNFα and interferon-γ [67], and by absolute and functional iron deficiency. Whereas iron is an essential nutrient and necessary for the formation of hemoglobin, iron therapy may affect leukocyte functions and cytokine production, promote oxidative stress and support bacterial growth. The killing capacity of PMNL isolated from CKD patients decreases in response to high-dose parenteral iron sucrose [68]. Therefore, atherosclerosis and infection may be long-term complications in which intravenous iron therapy in CKD patients plays an important role, especially in case of iron overload [69]. Moreover, iron therapy may not only adversely affect phagocytes, but also T and B lymphocytes in CKD patients [70]. 

Hepcidin, a peptide produced by the liver, is a regulator of iron distribution in the human body by affecting the flow of iron via binding to the cellular iron exporter ferroportin. The excessive production of hepcidin may lead to the relative deficiency of iron during inflammatory states causing anemia of inflammation characterized by a functional iron deficiency [71]. Hence, hepcidin represents a link between inflammation and anemia in CKD [72]. The elevated hepcidin levels in CKD have recently been suggested to be suppressed by EPO [73].

EPO, beyond its erythropoietic and cytoprotective effects, has immuno- modulatory properties [74]: EPO up-regulates TLR-4 in differentiating dendritic cells (DCs), rendering them more sensitive to stimulation by the TLR-4 ligand lipopolysaccharide.

### 5.2. Vitamin D, Calcium, Parathyroid Hormone and Fibroblast Growth Factor 23

The active vitamin D metabolite 1,25-dihydroxy-vitamin D3 (calcitriol) is not only synthesized in the kidney, but also in extra-renal tissues, e.g., activated monocytes/macrophages [75], and particularly in endothelial cells. In CKD, the synthesis of calcitriol is reduced. Both parathyroid hormone (PTH), the main stimulus of the rate-limiting enzyme 1alpha-hydroxylase, and hyperphosphatemia, the main inhibitory signal, are modified in CKD [76]. Uremic retention solutes may be responsible for changes in calcitriol production, resulting in calcitriol deficiency observed in renal failure [77]. The pleiotropic effects of vitamin D, such as modulation of the immune system, regulation of inflammatory responses and suppression of the renin-angiotensin system (see below Section 5.3) may slow down the progression of CVD [78]. Macrophage vitamin D receptor signaling may inhibit atherosclerosis in mice, partially by suppressing the local renin-angiotensin system [79].

Fibroblast growth factor 23 (FGF23) is secreted by osteoblasts in bone. FGF23 regulates the renal excretion and reabsorption of phosphate and reduces the production of 1,25-dihydroxy-vitamin D3. Elevated FGF23 concentrations are observed early in CKD and are suggested to be associated with increased mortality and disease progression [80]. A negative reciprocal relationship between FGF23 concentrations and declining renal function has been found in pediatric patients with pre-dialysis CKD Stages 3–5 [81]. In advanced CKD, FGF23 is strongly associated with all-cause mortality, cardiovascular events and initiation of chronic dialysis [82]. Since increased FGF23 plasma concentrations predict cardiovascular events in CKD patients, lowering FGF23 levels could be a target of novel therapeutic interventions in CKD [83,84]. An increase in [Ca^2+^]_i _is an important second messenger in PMNLs [55,56] involved in functional responses and the modulation of apoptosis [54,57,58]. In agreement with the literature [56,85,86,87], we observed increased basal levels of [Ca^2+^]_i_ in PMNLs from HD patients [50]. This increased basal [Ca^2+^]_i_ is associated with a decreased reactivity upon stimulation [6,88]. Bioincompatible membranes cause an increase in [Ca^2+^]_i_, whereas biocompatible membranes do not change [Ca^2+^]_i_ [86,89]. Dialysis treatment with biocompatible high-flux membranes can revert the increased [Ca^2+^]_i_ [50], suggesting a removal of factors responsible for the increased basal levels of [Ca^2+^]_i_. Differences in [Ca^2+^]_i_ have been observed between patients on EPO, and not on EPO [87]. Therefore, besides uremic retention solutes, EPO therapy may contribute to elevated [Ca^2+^]_i_ [86].

PTH levels are increased in CKD patients. Chronic excess of PTH in uremia affects PMNL functions via sustained elevation of their [Ca^2+^]_i_ [88]. Parathyroidectomy lowers, but does not normalize, PMNL [Ca^2+^]_i_ of CKD patients [85], further supporting the notion that other factors such as uremic retention solutes affect [Ca^2+^]_i _and functions of PMNLs. High levels of PTH in uremia also affect the metabolism and function of B cells [90], as well as *T*-lymphocyte functions contributing to changes in cellular immunity [91]. Furthermore, doses of calcitriol within the therapeutic range are able to induce changes in the secretion of cytokines (IL-1, IL-6 and TNF) of peripheral blood mononuclear cell from uremic patients [92].

### 5.3. Renin, Angiotensin

Renin is a circulating enzyme secreted by the kidney when blood pressure is low. It stimulates the production of angiotensin. The renin-angiotensin system plays a pivotal role in the regulation of blood pressure by modulating the vascular tone. The renin-angiotensin system is also involved in the pathogenesis of inflammation and the progression of CKD. T cells, natural killer cells and monocytes express the angiotensin receptor 1. T and natural killer cells also express angiotensin 2 and contain all renin-angiotensin system elements, suggesting that they are able to produce and deliver angiotensin 2 to sites of inflammation [93]. Therefore, lymphocyte activating activities of the renin-angiotensin system may lead to inflammation [94]. Angiotensin 2 also stimulates chemotaxis and may induce an inflammatory amplification system. The role of angiotensin 2 in stimulating phagocytes is further underlined by the evidence that it stimulates PMNL oxidative burst and increases cytosolic Ca^2+^ concentrations [95]. Furthermore, the susceptibility to T cell-mediated injury in anti-glomerular basement membrane antibody-induced glomerulonephritis is increased by local renin-angiotensin system activation, implying that drugs interfering with renin-angiotensin system could be useful in the treatment of immune renal diseases [96].

## 6. Uremic Toxins

**Figure 4:**
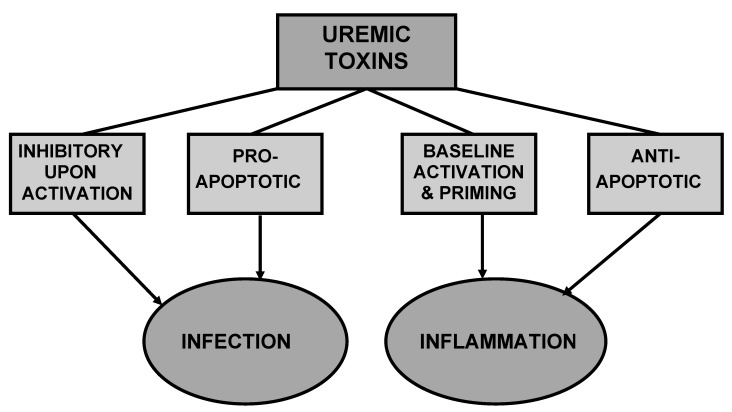
Different uremic toxins may exert antagonistic effects leading to infection and inflammation.

The retention of many compounds, which under normal conditions are filtered by the healthy kidneys, leads to the development of the uremic syndrome. Those retention solutes that interact negatively with biologic functions are called uremic toxins [97]. If they exert an inhibitory and/or pro-apoptotic effect on immune cells, these uremic toxins contribute to the susceptibility to infection (Figure 4), while culprits of baseline activation, priming and/or anti-apoptotic features give rise to inflammation. 

Uremic toxins playing an active role in vascular damage may also be generated or introduced into the body via the intestine [98]. In 2003, the European Uremic Toxin Work Group (EUTox; http://EUTox.info) composed an encyclopedic list of 90 uremic retention solutes known at that time [99]. Recently, this classification of normal and pathologic concentrations of uremic toxins has been extended and updated [100].

As a result of different hydrophobicity, low molecular weight organic substances may either exist in free water-soluble form or bind reversibly to serum proteins. Many of the currently known biological effects in uremic patients are attributed to protein-bound solutes. Their dialytic removal is largely hampered by their physicochemical properties. Therefore, alternative removal techniques, such as strategies to modify intestinal generation or absorption, are considered [101]. In CKD, proteins may exist in their native form or, as a result of exposure to the uremic milieu (Figure 3), become irreversibly changed by posttranslational modifications, resulting in altered structure and function. Examples are the heterogeneous groups of AGEs, AOPPs and carbamoylated proteins.

The identification and characterization of uremic toxins contributing to major uremia-related complications is a prerequisite for the critical evaluation and systematic design of preventive and therapeutic interventions for patients with CKD. Understanding the effects of uremic toxins will help to develop novel therapeutic strategies, such as improved removal of toxins, and the search for pharmacologic strategies blocking responsible pathophysiologic pathways [102]. *In vitro* assays testing the biologic effects of individual solutes represent a straightforward way to select candidates for further in-depth investigation. EUTox published basic protocols for the *in vitro* screening of uremic retention solutes, providing information about their availability, solubility and the appropriate preparation of stock solutions [103]. The use of the disease-relevant concentrations of solutes in *in vitro* assays is a precondition to obtaining relevant conclusions [104,105]. Functional disturbances caused by selected uremic toxins are summarized in Table 1.

**Table 1:** Functional disturbances caused by selected uremic toxins.

Uremic toxin	Functional disturbance
***LMW Solutes***	
Phenylacetic acid (PAA)	Macrophages: inducible nitric oxide synthase ↓ [106];
	PMNLs: oxidative burst, phagocytosis and integrin expression ↑; apoptosis ↓ [49]
Dinucleoside polyphosphates	Leukocytes: oxidative burst ↑ [107].
Guanidino compounds	Monocytes/macrophages: pro- and anti-inflammatory [108,109,110]
Indoxyl sulfate	Endothel: E-selectin ↑ [111]
*P*-cresyl sulfate	Leukocytes: basal oxidative burst ↑ [112]
Homocysteine (Hcy)	ICAM-1 ↑ [113]; damage of DNA [114] and proteins [115]
Methylglyoxal (MGO)	PMNLs: apoptosis ↑ [116], oxidative burst ↑ [117];
	Monocytes: apoptosis ↑ [118]
***Middle Molecules, Proteins***	
Immunoglobulin light chains (IgLCs)	PMNLs: chemotaxis ↓, glucose uptake stimulation ↓, glucose uptake basal ↑ [119]; apoptosis ↓ [47]
Retinol binding protein (RBP)	PMNLs: chemotaxis ↓, oxidative burst ↓, apoptosis ↓ [120]
Leptin	PMNLs: chemotaxis ↓, oxidative burst ↓ [121]
Resistin	PMNLs: chemotaxis ↓, oxidative burst ↓ [122]
Tamm-Horsfall protein (THP)	PMNLs: (high concentrations) apoptosis ↓, chemotaxis ↓, phagocytosis ↑; (low concentrations) chemotaxis ↑ [123]
High-density lipoprotein (HDL)	Loss of anti-inflammatory properties in uremia [124,125]
***Protein Modifications***	
Glucose-modified proteins	PMNLs: chemotaxis ↑, glucose uptake ↑, apoptosis ↑ [48]
AGE-modified albumin	Leukocytes: activating, pro- atherogenic [126]
AGEs	Macrophages: TNF and IL-1 secretion ↑ [127]
	Monocytes: Chemotaxis ↑ [128]
Glycated collagen	PMNLs: Adhesion ↑ [129]
Advanced oxidation protein products (AOPPs)	PMNLs and monocytes: oxidative burst ↑ [130]
Oxidized low-density lipoproteins (oxLDLs)	Macrophage activation [131];
	PMNLs and eosinophils: chemotaxis ↑, degranulation ↑ [132];
	Regulatory T cells: proteasome activity ↓ → cell cycle arrest and apoptosis [133]
Homocysteinylated albumin	Monocytes: adhesion ↑[134]

### 6.1. LMW Solutes

Phenylacetic acid (PAA) was identified as a novel uremic toxin in patients on regular HD [106]. PAA interferes with the expression of inducible nitric oxide synthase, which generates NO, a mediator of macrophage cytotoxicity [135]. Therefore, by inhibiting inducible nitric oxide synthase expression and reducing the cytotoxicity against intracellular bacteria, PAA may aggravate the immunodeficiency of CKD patients. PAA increases the activation of several PMNL functions, such as oxidative burst, phagocytosis and integrin expression, while it attenuates PMNL apoptotic cell death [49]. Hence, it may contribute to the inflammatory state in uremic patients, and consequently to increased cardiovascular risk. 

Dinucleoside polyphosphates are newly detected uremic retention solutes. Their pro-inflammatory properties—stimulation of the oxidative burst of PMNLs, monocytes and lymphocytes—may contribute to the development of atherosclerosis, probably in early CKD stages [107].

Guanidino compounds, such as guanidinopropionic acid and guanidinobutyric acid [108], methylguanidine, guanidine, guanidinosuccinic acid and guanidinoacetic acid [109] and symmetric dimethylarginine [110], exert pro-inflammatory, as well as anti-inflammatory, effects on monocyte/macrophage function, and thereby may contribute to the high prevalence of CVD and to the disposition to infection in CKD patients.

Both indoxyl sulfate and *p*-cresyl sulfate, two of the main protein-bound compounds, have a negative impact on the cardiovascular system and progression of kidney failure [101]. Indoxyl sulfate induces leukocyte-endothelial interactions through up-regulation of E-selectin [111]. Serum free *p*-cresyl sulfate levels predict cardiovascular and all-cause mortality in elderly hemodialysis patients [136]. The free, non-protein bound form of *p*-cresylsulphate predicts survival in CKD patients [137]. *P*-cresyl sulfate significantly increases the basal level of leukocyte oxidative burst activity, but does not affect the production of reactive oxygen species by stimulated leukocytes [112]. 

Hyperhomocysteinemia occurs in the majority of uremic patients undergoing HD treatment [138]. Hcy stimulates inter-cellular adhesion molecule-1 (ICAM-1) expression, leading to an increase in monocyte adhesion to endothelial cells, and consequently may create a proinflammatory environment in the vessel wall that initiates and promotes atherosclerotic lesion development [113]. Hcy can directly modify the expression of CD11b/CD18, CD14 and L-selectin on PMNLs, monocytes and lymphocytes, resulting in leukocyte adhesion and migration [113]. Hcy contributes to the genomic damage in CKD [114] and to the molecular damage of proteins, leading to clinical complications [115], such as accelerated atherogenesis [139].

Significant increases of plasma methylglyoxal (MGO) levels are a function of CKD stage. MGO accelerates PMNL apoptosis [116] and enhances the production of reactive oxygen species by PMNL [117]. MGO also induces monocytic apoptotic cell death, presumably via elevation of intracellular oxidant stress [118]. 

### 6.2. Middle Molecules, Proteins

IgLCs are synthesized by B cells slightly in excess of Ig heavy chains [140] in parallel to intact immunoglobulins. Therefore, they are found in the plasma of healthy people at low levels. Serum concentrations of free IgLCs are increased either by a diminished elimination, such as in patients with impaired kidney function, or as a result of an increased production, like in B-cell lymphoproliferative disorders, e.g., multiple myeloma. Polyclonal free IgLCs accumulate in the serum of patients with CKD, and their concentrations progressively increase with CKD stage [141]. Standard HD and HDF are not able to normalize their serum levels [142]. Extended HD with a protein-leaking dialyzer for patients with myeloma and renal failure is able to remove large amounts of free IgLCs [143]. IgLCs can affect PMNL functions. Free polyclonal IgLCs, isolated as monomers or dimers from uremic patients receiving HD treatment or undergoing continuous ambulatory peritoneal dialysis, significantly inhibit PMNL chemotaxis *in vitro *[119] and attenuate PMNL apoptosis [47]. The uptake of glucose is considered as a quantitative measurement of the state of activation of phagocytic cells. IgLCs reduce the stimulation of the PMNL glucose uptake, but stimulate its basal level. Therefore, free IgLCs contribute to pre-activation of PMNLs and interfere with the normal resolution of inflammation.

Retinol-binding proteins (RBPs) are a family of carrier proteins for retinol (vitamin A). Intracellular RBPs (1, 2, 5, and 7) have been found, e.g. in liver [144], parathyroid glands [145], epidermis [146] and small intestine [147]. RBP 3 is the interstitial form of this protein. RBP4, synthesized in the liver, is present in human plasma. RBP 4 and creatinine levels correlate in CKD patients [148]. In acute renal failure, the RBP serum concentration is increased as well [149]. RBP isolated from the ultrafiltrate of patients with acute renal failure interferes with PMNL chemotaxis, oxidative burst and apoptosis [120]. RBP4 is elevated in non-diabetic stage 5 CKD and correlates weakly with HbA1c and ApoA1, suggesting a role for RBP in the development of the uremic metabolic syndrome [150]. 

White adipose tissue is an active player in regulating immunity and inflammation [151]. Adipocytes have pluripotent signaling effects [152], and not only play a major role in cytokine, adipokine and chemokine secretion, but also in innate immunity [153]. The serum levels of adipokines, such as leptin and resistin, are increased in CKD [99,154,155]. This is not merely a result of decreased renal elimination, but also of increased production by adipocytes stimulated in the uremic milieu, e.g., by TNF-α [156]. Leptin reduces PMNL chemotaxis in a reversible manner and diminishes the stimulation of PMNL oxidative burst [121]. In humans, resistin is expressed primarily by macrophages in the visceral white adipose tissue [157]. It was also detected in PMNLs and monocytes [158]. Resistin concentrations are increased in sera of CKD patients [122,159,160,161]. It attenuates PMNL chemotaxis and decreases activation of PMNL oxidative burst [122]. Resistin is stored in PMNL granules, can be released upon challenge with inflammatory stimuli [162] and stimulates the chemotaxis of CD4-positve lymphocytes [163]. Hence, while PMNLs may decrease their own functions via resistin, they attract lymphocytes to the site of inflammation. 

Tamm-Horsfall protein (THP), also known as uromodulin, is a glycoprotein exclusively produced by the kidney in the distal loop of Henle and is a defense molecule against urinary tract infection [164]. GFR correlates positively with urinary THP, and negatively with serum THP [165]. In *in vitro* experiments, THP causes a dose-dependent increase in the secretion of pro-inflammatory cytokines from whole blood [165] and also influences several PMNL functions [123]. High THP concentrations found in the urine inhibit PMNL apoptosis and chemotaxis and stimulated PMNL phagocytosis, while low THP concentrations, which are observed in plasma increase PMNL chemotaxis [123]. These findings suggest a crucial immunomodulatory role of THP in host defense mechanisms of the urinary tract. The impact of THP on host immunity in the urinary tract is further highlighted by studies showing that THP activates myeloid dendritic cells via TLR-4 to acquire a fully mature dendritic cell phenotype [166]. Therefore, THP represents a link between the innate immune response and specific THP-directed cell-mediated immunity [166].

High-density lipoprotein (HDL) from healthy persons has anti-inflammatory properties. HDL and Apo A–I, its main protein component, significantly decrease CD11b surface expression on activated PMNLs [167] and monocytes [168] and reduce PMNL chemotaxis [167]. Apo A–I inhibits PMNL adhesion, oxidative burst and degranulation [169]. HDL from healthy individuals inhibits the production of inflammatory cytokines by peripheral monocytes, whereas HDL isolated from CKD patients did not show this anti-inflammatory feature [125]. Hence, uremia impairs the atheroprotective properties of HDL. The amount of serum amyloid A in the HDL particle from CKD patients inversely correlates with its anti-inflammatory potency [125]. HDL from CKD patients has also a reduced potential to inhibit the formation of monocyte chemoattractant protein-1, an important pro-inflammatory cytokine in early atherogenesis, in vascular smooth muscle cells [124].

### 6.3. Protein Modifications

In CKD, proteins may exist in their native form or, as a result of exposure to the uremic milieu, become irreversibly altered by posttranslational modifications. This leads to a changed structure and function, and consequently to cellular dysfunction and tissue damage. Enzyme activities, cofactors, hormones, low-density lipoproteins, antibodies, receptors and transport proteins may be affected.

#### 6.3.1. Advanced Glycation End-Products

In the presence of glucose, proteins are non-enzymatically modified. The resulting AGE formation leads to protein cross-linking [170]. Glycation is an unavoidable, minor feature of the physiological metabolism: 6% to 15% of human serum albumin is glycated in normal serum [171]. In CKD and diabetes, AGE levels are elevated as a result of decreased renal clearance and/or increased rate of glycation. In HD patients, AGE formation is enhanced by an increased oxidative stress, rather than by elevated glucose levels [172]. In uremic patients, the AGE level is even higher than in diabetic patients without renal disease [173]. 

Compared to unmodified proteins, proteins modified *in vitro* by glucose increase PMNL chemotaxis and the activation of PMNL glucose uptake [48]. PMNL apoptosis is enhanced in the presence of glucose-modified serum proteins. Albumin modified with specific AGE compounds has an activating, potentially pro- atherogenic effect on leukocyte responses [126]. AGEs stimulate TNFα and IL-1β secretion by peritoneal macrophages in PD patients, and thereby contribute to the altered permeability of the peritoneal membrane in long-term PD patients [127]. *In vitro*- and *in vivo*-formed AGEs are chemotactic for human monocytes, and sub-endothelial AGEs can initiate monocyte migration across an intact endothelial cell monolayer [128]. Glycation of collagen during uremia increases PMNL adhesion via the receptor of AGEs to collagen surfaces, and may thereby contribute to the inhibition of normal host defense in CKD patients [129].

#### 6.3.2. Oxidative Modifications

AOPPs, markers of phagocyte-derived oxidative stress, and uremic toxins with pro-inflammatory effects trigger the oxidative burst in PMNLs and monocytes [130]. In HD patients, AOPPs mainly result from MPO released by activated PMNLs, whereas the formation of AOPPs in predialysis patients primarily results from MPO-independent oxidation mechanisms [174]. Activated PMNLs can modify serum proteins via the production of reactive oxygen species. These modified proteins, in turn, bind to and activate PMNLs [175]. Therefore it has been suggested that local PMNL-initiated oxidative alterations of serum proteins may be a general autocrine and paracrine pro-inflammatory enhancer mechanism for PMNL activation and accumulation at the site of inflammation [175]. 

Oxidized low-density lipoproteins (oxLDLs) are main participants in the pathogenesis of atherosclerosis via binding and activating macrophages [131]. The protein moiety in oxLDLs is responsible for this activity [131]. OxLDLs can also stimulate chemotaxis and degranulation of both PMNLs and eosinophils [132]. OxLDLs inhibits proteasome activity in regulatory T cells, leading to cell cycle arrest and apoptosis, and as a result, to a dramatically decreased suppressive capacity of these cells [133].

Albumin, the most important antioxidant, can be fragmented in nephrotic patients, diabetics and ESRD patients due to a higher susceptibility to proteases induced by oxidative stress [176]. In turn, oxidation of albumin may contribute to the progression of oxidative stress in HD patients [177]. 

#### 6.3.3. Carbamoylation, Carbonylation and Homocysteinylation

A small percentage of urea that accumulates in the sera of uremic patients is converted to cyanate, causing a modification of serum proteins called carbamoylation. Carbamoylated molecules affect metabolic pathways, and may therefore contribute to uremic toxicity [178]. In CKD patients undergoing PD, carbamoylated proteins have been detected throughout the cytoplasm of PMNLs and monocytes, as well as on their cell surface [179]. 

Carbonylation is an irreversible modification caused by the introduction of carbonyl derivatives (aldehydes and ketones) into proteins. Chronic uremia is associated with an increased carbonyl overload (“carbonyl stress”) targeting several different plasma proteins. Carbonylated albumin displays biological effects that may be relevant to uremic atherosclerosis [180].

Levels of homocysteinylated proteins are elevated in hemodialysis patients. Treatment with homocysteinylated albumin specifically increases monocyte adhesion to endothelial cells [134].

## 7. Further Aspects of Immune Dysfunction in Uremia

### 7.1. Antigen-Presenting Cells

APCs present the antigen together with the major histocompatibility complex II and can be DCs, monocytes, or in special cases, B-cells. Pre-activated APCs contribute to the malnutrition-inflammation-atherosclerosis syndrome and may also affect T-cell functions. This derangement may result from an impaired interaction between the APCs and the *T*-lymphocytes [10]. 

DCs are essential for innate and adaptive immunity. Monocyte and monocyte-derived DC functions are disturbed in HD patients. In CKD stage IV patients, the terminal differentiation of monocyte-derived DCs is impaired [181]. DCs from normal persons cultured in uremic sera and DCs of HD patients cultured in normal or uremic sera show decreased endocytosis and impaired maturation, suggesting the involvement of soluble and intrinsic factors [182]. 

Kidney DCs bind glomerular antigens and present them to infiltrating T cells, leading to the production of proinflammatory cytokines and activation of further immune effector cells, main components of the well-known tubulointerstitial mononuclear infiltrate characteristic of progressive renal disease [183]. Therefore, effector T cell dysregulation by intra-renal DCs may represent a so-far unknown mechanism by which glomerular damage results in chronic tubulointerstitial inflammation.

DCs as the most effective APCs are crucial for the initiation of immune responses, including acute and chronic allograft rejection. The compound FK778 is an inhibitor of DNA replication and tyrosine kinases and acts as a strong immunosuppressant preventing chronic allograft rejection by inhibiting the activation and function of DCs [184]. The immunosuppressive action of FK778 may be mediated by blocking the formation of the immunological synapse [185].

### 7.2. Epigenetics

Epigenetics is the study of changes in gene expression that occur without changes in DNA sequence. The significance of epigenetics in CKD has been recognized only within the last few years. Complex interactions between aberrant DNA methylation and uremic dysmetabolism contribute to the development of premature uremic vascular disease [186]. Causes of genomic damage are chronic cell activation related to HD treatment and oxidative stress induced by uremic toxins, such as AGEs, and hyperhomocysteinemia. Inflammation, dyslipidaemia, hyperhomocysteinaema, oxidative stress, as well as vitamin and nutritional deficiencies may affect the epigenome [187].

Chronic activation of immunocompetent cells leads to stress-induced premature senescence, which is characterized by a decrease in telomere length. In CKD, immunocompetent cells, such as mononuclear cells and lymphocytes, undergo stress-induced premature senescence associated with chronic cell activation, and thereby may contribute to the chronic inflammatory state of CKD patients [188].

The genomic damage of peripheral lymphocytes increases with declining kidney function as a result of uremia and a state of genomic instability, caused by individual genetic factors [189]. Dialysis treatment, *per se*, is a potential source of damage and may be responsible for the T-cell specific immunodeficiency correlated with uremia [190]. DNA damage by AGEs may also be important in the development of several forms of cancer, a disease with increased occurrence in CKD patients [191]. 

There is a correlation between Hcy plasma concentration and genomic damage in lymphocytes. A reduction of Hcy levels by supplementation with folic acid and vitamin B12 in dialysis patients leads to a reduction in genomic damage in peripheral blood leukocytes [114]. Via the metabolic precursor of homocysteine, *S*-adenosylhomocysteine, a powerful methyltransferase competitive inhibitor, hyperhomocysteinemia leads to DNA hypomethylation [192]. DNA hypomethylation is found in the mononuclear cell fraction of uremic patients with hyperhomocysteinemia [115]. However, the genotoxic effect is limited to high Hcy concentrations, suggesting that the DNA-damaging effect of Hcy in CKD patients is only conceivable upon local Hcy accumulation [193].

### 7.3. Antineutrophil Cytoplasmic Autoantibodies

Antineutrophil cytoplasmic autoantibodies (ANCAs) are autoantibodies against PMNL autoantigens, such as PMNL granule protein proteinase 3 and MPO. Whereas in CKD patients no correlation with primary renal diseases or dialysis membrane materials was found, a higher incidence was detected in patients undergoing HDF. Backfiltration of contaminated dialysate may induce ANCAs via an increased cytokine generation [194]. Complement participation is required in the pathogenesis of ANCA-induced necrotizing crescentic glomerulonephritis (NCGN). The anaphylatoxin C5a is crucial to disease induction via the PMNL C5a receptor [195]. Therefore, C5a and the PMNL C5a receptor may compose an amplification loop for ANCA-mediated PMNL activation. Furthermore, the phosphoinositol-3-kinase-gamma isoform plays a pivotal role in ANCA-induced NCGN, and represents a potential novel treatment target [196]. In patients developing primary small vessel vasculitis, primed circulating PMNLs more readily undergo apoptosis accompanied by the surface expression of protein proteinase 3 and MPO, targets for ANCAs [197]. *In vitro*, ANCAs stimulate primed PMNLs to degranulate and generate reactive oxygen species, which in turn can trigger apoptosis [197].

## 8. Conclusions

Cardiovascular disease (CVD) and infections are directly or indirectly associated with a disturbed immune response and account for the high incidence of morbidity and mortality among patients with kidney dysfunction. Besides uremic toxins accumulating in CKD patients as a result of impaired glomerular filtration, deranged renal metabolic activities interfere with the immune defense in uremia.
